# Thyroid gland visualization with 3D/4D ultrasound: integrated hands-on imaging in anatomical dissection laboratory

**DOI:** 10.1007/s00276-016-1775-x

**Published:** 2016-12-01

**Authors:** John L. Carter, Ankura Patel, Gabriel Hocum, Brion Benninger

**Affiliations:** 1 Medical Anatomy Center, Western University of Health Sciences, COMP-Northwest, 200 Mullins Drive, Lebanon, OR 97355 USA; 2 Medical Anatomy Center, Department of Medical Anatomical Sciences, Western University of Health Sciences, COMP-Northwest, Lebanon, USA; 3 Department of Orthopedic Surgery, General Surgery, and Sports Medicine, Samaritan Health Services, Corvallis, OR USA

**Keywords:** 3D ultrasound, 3D/4D ultrasound, Ultrasonography, Gross anatomy, Thyroid gland, First-year medical students, Medical education

## Abstract

**Purpose:**

In teaching anatomy, clinical imaging has been utilized to supplement the traditional dissection laboratory promoting education through visualization of spatial relationships of anatomical structures. Viewing the thyroid gland using 3D/4D ultrasound can be valuable to physicians as well as students learning anatomy. The objective of this study was to investigate the perceptions of first-year medical students regarding the integration of 3D/4D ultrasound visualization of spatial anatomy during anatomical education.

**Methods:**

108 first-year medical students were introduced to 3D/4D ultrasound imaging of the thyroid gland through a detailed 20-min tutorial taught in small group format. Students then practiced 3D/4D ultrasound imaging on volunteers and donor cadavers before assessment through acquisition and identification of thyroid gland on at least three instructor-verified images. A post-training survey was administered assessing student impression.

**Results:**

All students visualized the thyroid gland using 3D/4D ultrasound. Students revealed 88.0% strongly agreed or agreed 3D/4D ultrasound is useful revealing the thyroid gland and surrounding structures and 87.0% rated the experience “Very Easy” or “Easy”, demonstrating benefits and ease of use including 3D/4D ultrasound in anatomy courses. When asked, students felt 3D/4D ultrasound is useful in teaching the structure and surrounding anatomy of the thyroid gland, they overwhelmingly responded “Strongly Agree” or “Agree” (90.2%).

**Conclusion:**

This study revealed that 3D/4D ultrasound was successfully used and preferred over 2D ultrasound by medical students during anatomy dissection courses to accurately identify the thyroid gland. In addition, 3D/4D ultrasound may nurture and further reinforce stereostructural spatial relationships of the thyroid gland taught during anatomy dissection.

## Introduction

Visualizing structural anatomy of the human body is essential for building a strong clinical knowledge base, gaining fundamental understanding of diagnostic and treatment skills. Contemporary anatomy courses provide visuals through imaging and illustrations during didactic lectures along with guided dissection of cadavers within the anatomy laboratory. At the hands of novice dissectors, anatomical structures can easily become disfigured, preventing students from having the opportunity to appreciate structures and surrounding anatomy. An alternative to the classical human body dissection is to provide a prosection to the students, but this may deprive students of the benefits gained from hands-on experience while learning about the architecture of the human body which kinesthetic learners thrive on. In recent years, there have been improved efforts to include clinical imaging modalities to assist students in clinically based anatomy.

Images from X-ray, CT, and MRI images are commonly included during didactic anatomy lectures, though the actual imaging modalities may not be accessible for use by students. There has been exploration of integrating a hands-on ultrasound (US) experience within anatomy education during the past 20 years [12] yielding novel teaching methods and technology, such as using Google Glass in conjunction with ultrasound utilization in anatomy education [1]. Dreher and colleagues [5] have previously shown that ultrasound imaging can be implemented during an anatomy course for first-year medical students with students reporting enhanced understanding of anatomy coupled with an increased interest in ultrasonography. Similarly, Brown and colleagues [2] reported nearly all (96%) of first-year medical students who utilize ultrasound in their gross anatomy curriculum agree that ultrasound-based teaching increased their knowledge of anatomy previously acquired through traditional teaching methods. Furthermore, through utilization of ultrasound imaging within anatomy laboratories, the teaching of living anatomy becomes an option to further students’ clinical and anatomical knowledge [3, 6]. Despite the enthusiasm to incorporate the use of ultrasonography into anatomy education, one limitation is the ability to appreciate the structures and its surroundings in three dimensions; this, however, can be resolved through the use of 3D/4D ultrasound (4D denotes three-dimensional imaging rendered in real time).

Modern 3D US was first developed in 1986 and utilized with the goal of visualizing a live fetus in utero. A 3D probe and US scanner system was made commercially available in 1989, and since has been widely used in obstetrics [7]. In addition, the acquisition of 3D US images can provide tremendous clinical value in cases inclusive of but not limited to: guiding a procedure, where visualizing surrounding structures is of importance, such as administering nerve blocks [4] and making a volumetric assessment of internal organs [9].

The thyroid gland is part of the endocrine system, and multiple clinical pathologies can be revealed through the use of ultrasound imaging. Presentations of thyroid gland pathology that can be detected using ultrasound include enlarged glands as well as nodule growth on the gland itself. 3D US has been previously demonstrated to be useful in visualizing the thyroid gland to reveal pathology [8, 10, 11] and to make volumetric measurements [9]. The objective of this study was to assess whether a 3D/4D US probe can be used to identify the thyroid gland of cadavers and on live subjects by first-year medical students and to elucidate student perception of their experience using 3D/4D US.

## Materials and methods

A literature search was performed using Google Scholar regarding 3D/4D US probe use within an anatomy laboratory to identify the thyroid gland of cadavers and on live subjects by medical students during anatomy courses. Institutional Review Board approval was received for this project (#12/IRB/003), and informed consent was provided to all participants. All 108 students enrolled in the medical anatomy course participated in learning 3D/4D ultrasound imaging of the thyroid gland and 92 (85.2%) responded to the voluntary post-training survey.

Initially through readings and didactic lectures, students were taught regarding the structure, blood supply, and lymphatics of the thyroid gland, as well as the head and neck. This knowledge was supplemented with dissection, allowing for in depth view and exploration of the thyroid gland and related structures. Finally, students were allowed to apply and expand their understanding with ultrasound training.

Students were taught in a single small group session (4–8 students) lasting approximately 20 min. Teaching sessions included demonstration of a proper probe placement, usage of the ultrasound system (Fukuda Denshi UF-760-AG ultrasound system outfitted with a 3D/4D US probe), imaging of the thyroid gland using 2D and 3D/4D US, and accurate identification of the lobes of the thyroid gland as well its relationship with discernable surrounding anatomical landmarks. Students then practiced ultrasound techniques on live subjects and/or cadavers. Student technique using the ultrasound system and image acquisition was monitored by the instructors of the teaching sessions to ensure the quality and proper interpretation of the 3D/4D images. Each student acquired and interpreted at least three 3D/4D images of the thyroid gland. Upon completion of the session, participants filled out a brief survey on their experience and if they felt the session was helpful in facilitating their learning of anatomy. The post-training survey utilized a five-point Likert scale on each question to help gauge student perceptions about their experience with applying 3D/4D US in assisting their educational experience.

## Results

**Fig. 1:**
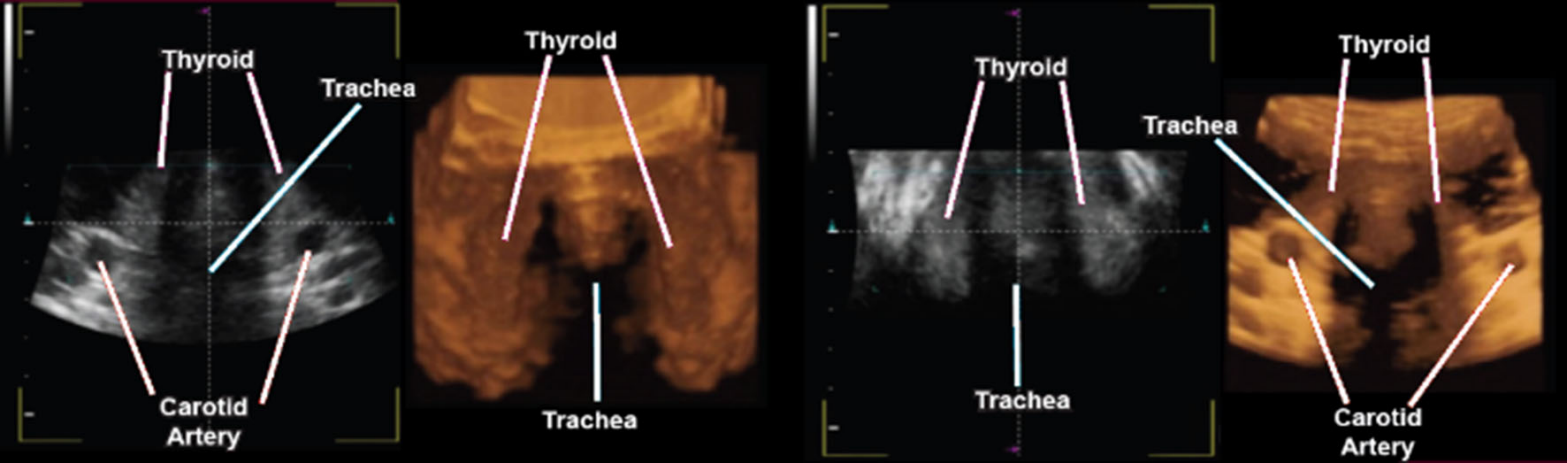
Sample of student obtained images. In addition to the thyroid gland, major anatomical landmarks were identified in both 2D and 3D/4D US imaging

**Fig. 2:**
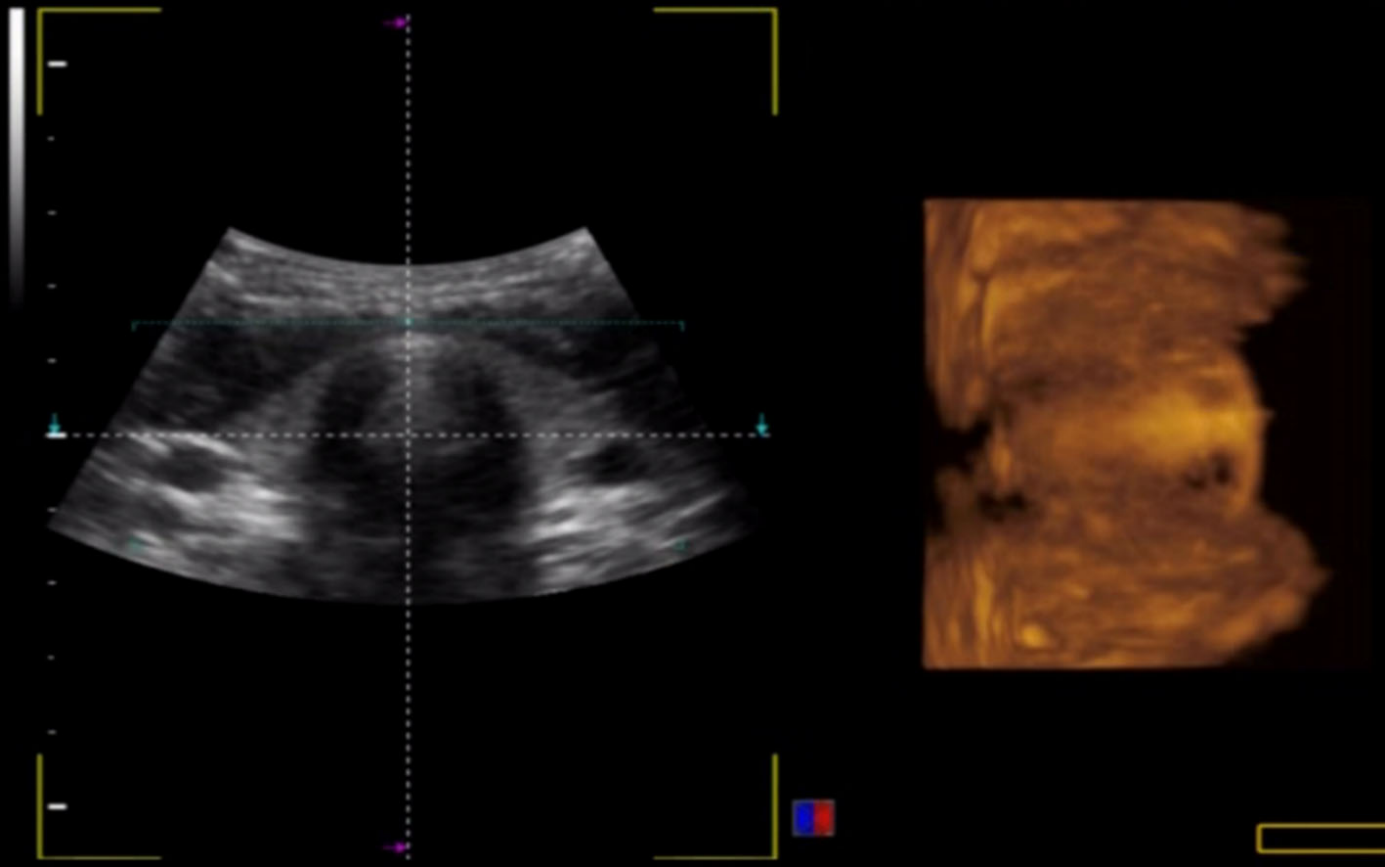
Example of an acquired image by students. Corresponding raw 2D (*left*) and 3D (*right*) ultrasound images were displayed simultaneously. The real-time 3D/4D image is displayed on an orthogonal plane relative to the 2D image

**Fig. 3:**
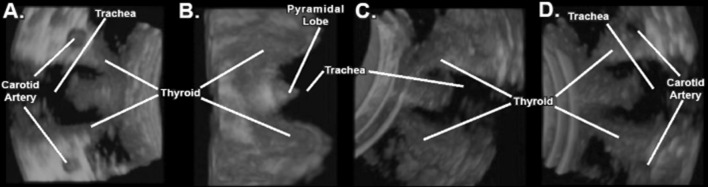
Images acquired via 3D/4D US are easily rotated, allowing for further investigation of the thyroid gland and its surrounding anatomy. **a** Initial probe placement has a limited* top–down *view of the thyroid gland. **b** 90° vertical rotation allows for a horizontal view and allows for better appreciation of the stereo-structure of the thyroid gland, including the pyramidal lobe. **c** 3D/4D US images can be rotated 360° in all three axes, allowing for oblique views. **d** 180° vertical rotation of the thyroid gland. The ability of 3D/4D US images to rotate in all axes allows for a comprehensive view of the thyroid gland without exposure to ionizing radiation

Literature search revealed no known use of 3D/4D ultrasound by medical students as part of medical anatomy courses. Of the 108 first-year medical students invited to participate in this study, 92 students (83.6%) with an age range of 21–43 years participated. All students demonstrated a proper use of the 3D/4D probe and identified major anatomical landmarks in addition to the thyroid gland (Fig. 1) while under the observation of instructors. During 3D/4D US imaging of the thyroid gland, students explored the different viewing angles and contours of the thyroid gland as the ultrasound system is capable of displaying both 2D and 3D/4D images simultaneously, allowing for a comparison and observation of anatomical landmarks on different spatial planes (Figs. 2, 3). Student perception of their experience with 3D/4D ultrasound was determined using a six question survey utilizing a 5-point Likert scale to help quantify student responses (Table 1).

**Table 1:**
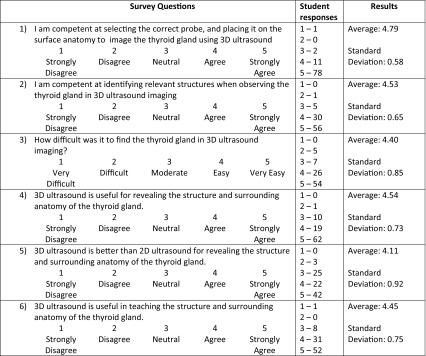
Questions included in the post-training survey and corresponding student responses (*n* = 92)

Nearly, all the students (96.7%) agreed or strongly agreed they felt competent at selecting the correct ultrasound probe and placing it correctly on the surface anatomy with student response averaging 4.79 on the five-point Likert scale (1—Strongly Disagree, 5—Strongly Agree). When students were asked if they felt competent at identifying relevant structures when observing the thyroid gland in 3D/4D ultrasound imaging, the response was positive with 86 students (93.5%) strongly agreed or agreed with an average of 4.53. To gauge the perceived difficulty of the 3D/4D ultrasound imaging, students were asked about the difficulty of finding the thyroid gland in 3D/4D ultrasound imaging to which 87.0% rated the experience “Easy” or “Very Easy” with an overall average of 4.40 on the Likert scale (1—Very Difficult, 5—Very Easy).

The last three questions aimed to explore the perceived usefulness of 3D/4D ultrasound in learning anatomy. A majority of students (88.0%) either agreed or strongly agreed 3D/4D ultrasound is useful for revealing the thyroid gland and surrounding structures. In comparison with 2D ultrasound, 69.6% of students agreed 3D/4D ultrasound is better at revealing the structure and surrounding anatomy of the thyroid gland. Furthermore, the average of responses for the comparison between 2D and 3D/4D ultrasound was 4.11 on the five-point Likert scale, giving that the overall student perception 3D ultrasound is better than 2D ultrasound in the study of structural anatomy. Finally, students were asked if they felt that 3D/4D ultrasound is useful in teaching the structure and surrounding anatomy of the thyroid gland. Students overwhelmingly responded “Strongly Agree” or “Agree” with 83 corresponding student responses (90.2%) and an average of 4.45 of 5 on the Likert scale.

## Discussion

3D/4D ultrasound imaging helps to provide users with more information and visualization of the architecture and orientation of internal organs as compared to 2D ultrasound. As experienced by the students, the 3D/4D ultrasound images can be rotated 360° in all three major axes allowing for a more comprehensive view of the thyroid gland (Fig. 3). This hands-on interaction with probe time coupled with the autonomy of choosing the viewing angle of the structures may have contributed to the positive student experiences.

**Fig. 4:**
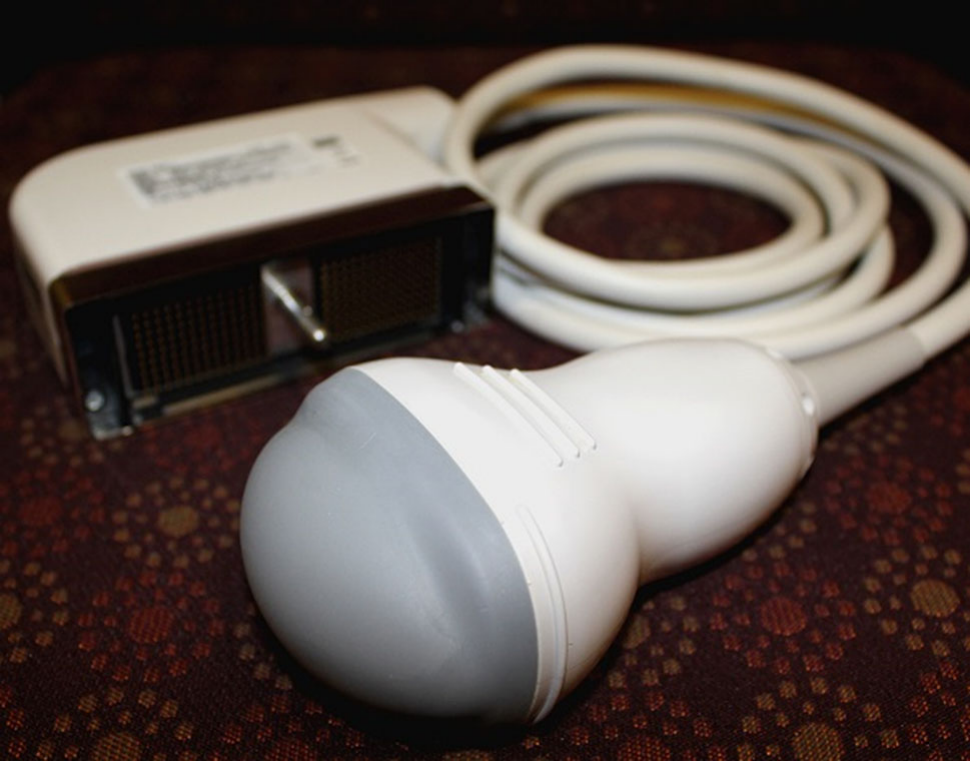
Fukuda Denshi 3D/4D ultrasound probe

With regard to students selecting the correct probe and placing it accurately on the surface anatomy, the 3D/4D probe has a characteristic size and shape (Fig. 4) as compared to other types of ultrasound probes. For the best visualization of the thyroid gland, the placement of the probe was superior to the jugular notch and rested on the clavicles with slight feathering to obtain a useful image of the thyroid gland. After demonstrating use of the 3D/4D US, students were quick to replicate the proper placement and imaging. This resounded through the response of 96.7% of students feeling that they are competent at selecting the correct probe and properly placing it to image the thyroid gland. In addition, 87.0% of the students who participated were able to identify the thyroid gland in 3D/4D ultrasound imaging and felt the experience was “Very Easy” or “Easy”. This demonstrates that integration of 3D/4D ultrasound into the anatomy laboratory to help facilitate medical anatomy education can be implemented easily.

Students believed that (1) 3D/4D ultrasound was useful in depicting the structure of the thyroid gland, (2) 3D/4D ultrasound was superior in revealing the structure and surrounding anatomy of the thyroid gland when compared to 2D ultrasound, and (3) overall, 3D/4D ultrasound is useful in teaching the thyroid gland and its surrounding anatomy. Given the inherent differences between 2D and 3D/4D ultrasound imaging coupled with the specific architecture of the thyroid gland, it is understandable why students favor 3D/4D ultrasound usage. Through 3D/4D imaging, students were able to identify both lobes of the thyroid gland, and in some cases, were able to visualize the normal variant of the presence of the pyramidal lobe. Furthermore, the authors of this study believe that integration of 3D/4D ultrasound imaging helps students identify the location of the thyroid gland, allowing for better understanding of hand placement and palpation of the thyroid gland during clinical physical examinations.

**Fig. 5:**
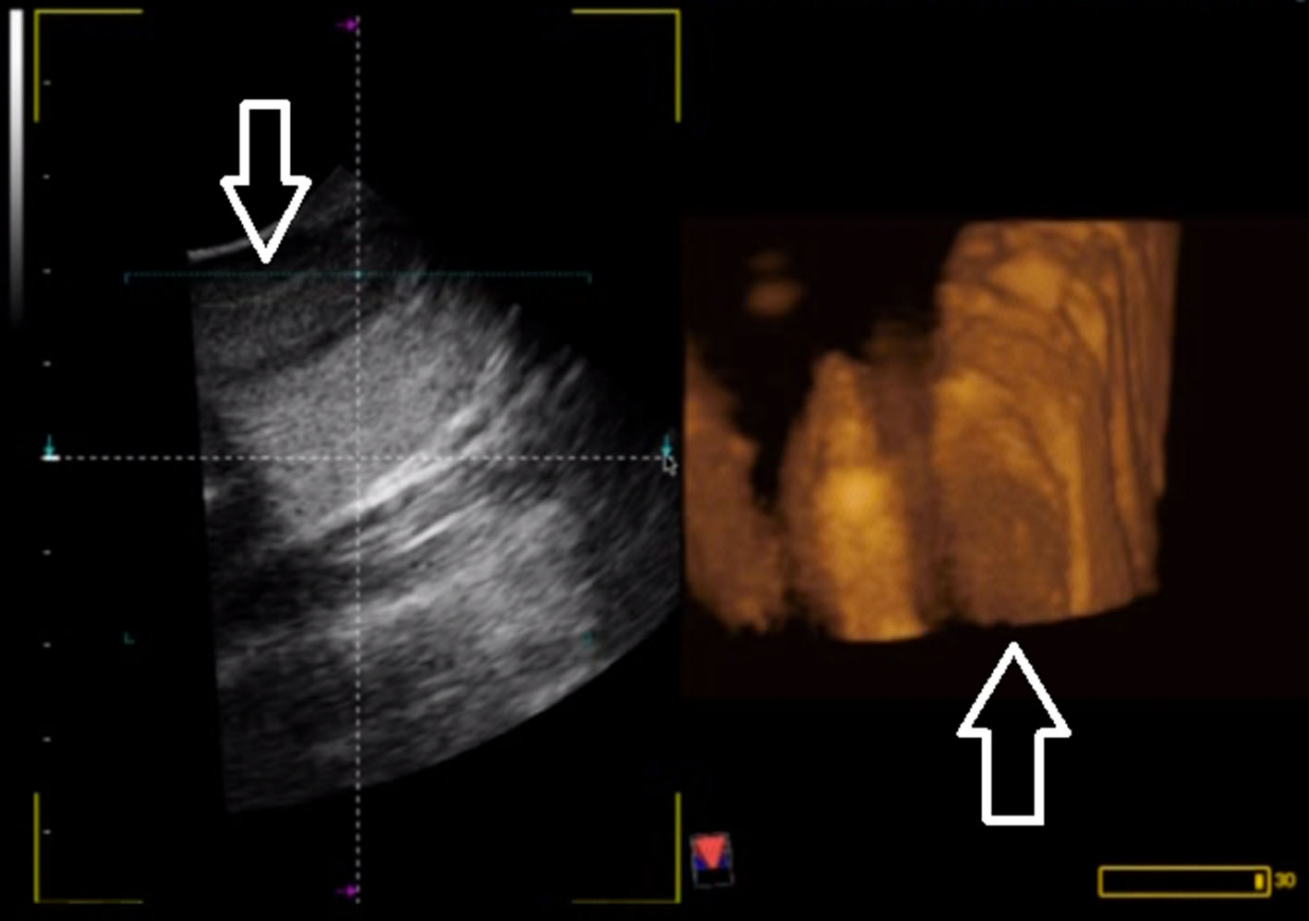
Incidental nodule found during ultrasound training. 2D ultrasound was used initially, as the thyroid nodule was discovered (*left*). Imaging using 3D/4D ultrasound (*right*) depicts the nodule and allows to see the nodule with both height and width from this viewpoint. The 3D/4D image can be rotated to further investigate the depth of the nodule, allowing for a better understanding of the size and shape of the nodule

For those intending to integrate ultrasound into anatomy courses, when imaging of the thyroid gland, there exists a potential to identify previously unknown pathology. Due to the chance that this may occur, a disclaimer must be provided to the students about this risk. In addition, participation to have a student’s thyroid gland imaged should be voluntary due to potentially revealing sensitive medical information. During this study, as students were practicing ultrasound techniques and imaging on other students, there were instances of incidentally discovering thyroid nodules. Within the educational setting, it can be demonstrated how measurements can be made on the nodules and how the 3D/4D image allowed for a more comprehensive view of potential pathologies (Fig. 5).

Limitations of this study include only encompassing one class at one medical school. Further investigation is required to gain better perspective on the benefits of integrating 3D/4D ultrasound usage in the teaching of anatomy. However, with all students being able to correctly identify the thyroid gland in cadavers and student volunteers only after a single training session, performing of 3D/4D US imaging and interpretation of 3D/4D US images have been shown to be manageable by first-year medical students. Through the use of 3D/4D US, students receive hands-on, clinically relevant experience at imaging internal organs within the human body. The deeper understanding of the stereostructural anatomy attained by students can be clinically relevant in the practice of medicine as to explain symptoms, interpret radiology, assess treatment, and prepare for surgery.

## Conclusion

This study revealed that a 3D/4D ultrasound probe can be successfully used and welcomed by medical students during anatomy courses. 3D/4D US has the potential to be a powerful learning tool supplementing the traditional anatomy lab by elucidating the stereostructural relationships of internal organs and limbs. In the context of a medical anatomy laboratory, utilization of 3D/4D ultrasound may help facilitate the learning of structural anatomy of the thyroid gland and subsequent interpretation of 3D CT/MRI imaging.

## References

[1] Benninger B (2015). Google Glass, ultrasound and palpation: the anatomy teacher of the future?. Clin Anat.

[2] Brown B, Adhikari S, Marx J, Lander L, Todd GL (2012). Introduction of ultrasound into gross anatomy curriculum: perceptions of medical students. J Emerg Med.

[3] Carter JL, Hocum G, Pellicer R, Patel A, Benninger B (2016). Integration of 3D/4D ultrasound in teaching medical anatomy. Med Sci Educ.

[4] Clendenen NJ, Robards CB, Clendenen SR (2014). A standardized method for 4D ultrasound-guided peripheral nerve blockade and catheter placement. Biomed Res Int.

[5] Dreher S, DePhilip R, Bahner D (2014). Ultrasound exposure during gross anatomy. J Emerg Med.

[6] Jurjus R, Dimorier K, Brown K, Slaby F, Shokoohi H, Boniface K, Liu Y (2014). Can anatomists teach living anatomy using ultrasound as a teaching tool?. Anat Sci Educ.

[7] Kurjak A, Chervenak F (2011). Donald school textbook of ultrasound in obstetrics and gynecology.

[8] Markova E, Bashilov V, Zubarev A (2007) 3D US is a base for virtual surgery in thyroid mass. Samsung Medison March 9, 2007 Ed. 1-4

[9] Nurunnabi AS, Ara S, Jahan MU (2012). A postmortem study on the volume of the human thyroid gland. Bangladesh Med Res Counc Bull.

[10] Slapa RZ, Jakubowski WS, Slowinska-Srzednicka J, Szopinski KT (2011). Advantages and disadvantages of 3D ultrasound of thyroid nodules including thin slice volume rendering. Thyroid Res.

[11] Slapa RZ, Slowinska-Srzednicka J, Szopinski KT, Jakubowski W (2006). Gray-scale three-dimensional sonography of thyroid nodules: feasibility of the method and preliminary studies. Eur Radiol.

[12] Teichgräber UK, Meyer JM, Poulsen Nautrup C, von Rautenfeld DB (1996). Ultrasound anatomy: a practical teaching system in human gross anatomy. Med Educ.

